# Next-generation sequencing for pediatric CNS tumors: does it add value in a middle-income country setup?

**DOI:** 10.3389/fonc.2024.1329024

**Published:** 2024-02-19

**Authors:** Nisreen Amayiri, Maysa Al-Hussaini, Bayan Maraqa, Shaza Alyazjeen, Qasem Alzoubi, Awni Musharbash, Ahmad Kh. Ibrahimi, Nasim Sarhan, Mouness Obeidat, Cynthia Hawkins, Eric Bouffet

**Affiliations:** ^1^ Department of Pediatrics, King Hussein Cancer Center, Amman, Jordan; ^2^ Department of Pathology and Laboratory Medicine, King Hussein Cancer Center, Amman, Jordan; ^3^ Department of Molecular Laboratory, King Hussein Cancer Center, Amman, Jordan; ^4^ Department of Diagnostic Radiology, King Hussein Cancer Center, Amman, Jordan; ^5^ Department of Surgery, King Hussein Cancer Center, Amman, Jordan; ^6^ Department of Radiation Oncology, King Hussein Cancer Center, Amman, Jordan; ^7^ Department of Pediatric Laboratory Medicine, The Hospital for Sick Children, Toronto, ON, Canada; ^8^ Division of Hematology/Oncology, The Hospital for Sick Children, Toronto, ON, Canada

**Keywords:** next-generation sequencing (NGS), children, CNS tumors, low-middle-income countries (LMIC), targeted therapy, compassionate access

## Abstract

**Introduction:**

Advances in molecular diagnostics led to improved targeted interventions in the treatment of pediatric CNS tumors. However, the capacity to test for these is limited in LMICs, and thus their value needs exploration.

**Methods:**

We reviewed our experience with NGS testing (TruSight RNA Pan-Cancer-seq panel) for pediatric CNS tumors at KHCC/Jordan (March/2022–April/2023). Paraffin blocks’ scrolls were shipped to the SickKids laboratory based on the multidisciplinary clinic (MDC) recommendations. We reviewed the patients’ characteristics, the tumor types, and the NGS results’ impact on treatment.

**Results:**

Of 237 patients discussed during the MDC meetings, 32 patients (14%) were included. They were 16 boys and 16 girls; the median age at time of testing was 9.5 years (range, 0.9–21.9 years). There were 21 samples sent at diagnosis and 11 upon tumor progression. The main diagnoses were low-grade-glioma (15), high-grade-glioma (10), and other histologies (7). Reasons to request NGS included searching for a targetable alteration (20) and to better characterize the tumor behavior (12). The median turnaround time from samples’ shipment to receiving the results was 23.5 days (range, 15–49 days) with a median laboratory processing time of 16 days (range, 8–39 days) at a cost of US$1,000/sample. There were 19 (59%) tumors that had targetable alterations (FGFR/MAPK pathway inhibitors (14), checkpoint inhibitors (2), NTRK inhibitors (2), and one with PI3K inhibitor or IDH1 inhibitor). Two rare BRAF mutations were identified (BRAFp.G469A, BRAFp.K601E). One tumor diagnosed initially as undifferentiated round cell sarcoma harbored NAB2::STAT6 fusion and was reclassified as an aggressive metastatic solitary fibrous tumor. Another tumor initially diagnosed as grade 2 astroblastoma grade 2 was reclassified as low-grade-glioma in the absence of MN1 alteration. NGS failed to help characterize a tumor that was diagnosed histologically as small round blue cell tumor. Nine patients received targeted therapy; dabrafenib/trametinib (6), pembrolizumab (2), and entrectinib (1), mostly upon tumor progression (7).

**Conclusion:**

In this highly selective cohort, a high percentage of targetable mutations was identified facilitating targeted therapies. Outsourcing of NGS testing was feasible; however, criteria for case selection are needed. In addition, local capacity-building in conducting the test, interpretation of the results, and access to “new drugs” continue to be a challenge in LMICs.

## Introduction

Over the last decade, several advancements, particularly next-generation sequencing (NGS) and DNA methylation profiling, improved our understanding of CNS tumors (1). As a result, a refined classification of CNS tumors leads to the integration of the molecular diagnosis in the recent 2021 WHO-CNS tumors classification (CNS-5) (2). This should help in a better prediction of tumors’ prognosis allowing to tailor therapy accordingly. Identifying potentially actionable alterations would, theoretically, result in utilizing targeted therapies for a better control of tumor growth. In the INFORM registry (3), where most tumors were refractory or relapsed solid tumors, 446 of the 519 patients (85.9%) had at least one actionable target. Eventually, 147 patients (28%) received a matched targeted drug whether through clinical trials, off-label use programs, or compassionate use programs. Matched targeted therapy with ALK, NTRK, and BRAF inhibitors showed significantly improved progression-free survival (PFS, p = .012) and overall survival (OS, p = .036) in comparison with conventional treatment or no treatment (4). These longer PFS and OS were also found in a comprehensive literature review on the clinical impact of NGS tests for the management of adults with advanced cancer (5).

Targeted panel-based NGS, like TruSight, is designed to sequence multiple selected cancer genes to allow for a rapid turnaround time using a small amount of tissue. A negative NGS result either means that the tumor has no detectable molecular alteration or this might be related to low tumor cellularity. Expertise is needed in interpreting the NGS results and integrating them with the histological findings to maximize its diagnostic and prognostic yield (6). This may help personalize the management of individual patients by early introduction of targeted interventions for aggressive tumors. With a more comprehensive DNA and RNA sequencing, germline mutations may be detected with further implications on the care provided to patients through counseling and cancer screening (3, 7).

The addition of the molecular layer of diagnosis to the CNS-5 classification (2) remains a challenge to many low–middle-income countries (LMICs). While surrogate immunohistochemistry (IHC) is relatively available and cheap, it does not cover the full range of the targetable mutations, and its interpretation remains subjective. The use of technologies like NGS and DNA methylation profiling is far more innovative with the need for a technical infrastructure and advanced personnel training. In a Korean pilot study (8) to evaluate the preliminary efficacy and clinical feasibility of NGS-based targeted anticancer therapy in refractory solid tumors, Moon et al. (8) found that 41.7% of patients did not start the targeted therapy due to a decline in their performance status, 20.8% due to stable disease with a previous treatment, and 16.7% due to lack of access to the targeted medication. They encountered several obstacles in their study; NGS was an outsourced test sent to the United States with a turnaround time of 4 weeks, in addition to the lack of insurance coverage for the NGS cost, and the limited access to the targeted medications. Similar data on the NGS utilization from LMICs are limited.

King Hussein Cancer Center (KHCC) is the only cancer-dedicated hospital treating children and adults in Jordan. It has a well-established pediatric neuro-oncology service and multidisciplinary clinic (MDC) team since 2003 with a twinning program with the Hospital for Sick Children (SickKids) in Toronto (9). More than 70% of the Jordanian children with CNS tumors are treated in this service in addition to consultations for non-Jordanians (with a total of 80–110 newly diagnosed cases per year). With the increasing role of molecular and sequencing information in the management of pediatric brain tumors and the implementation of the CNS-5 classification, discussions during the monthly teleconferences between KHCC and SickKids progressively involved the potential benefit of assessing molecular tumor alterations to help us reach the appropriate diagnosis in challenging cases or consider some targeted therapies in some patients. In this context, an outsourcing testing approach was agreed on. We collaborated with the SickKids Clinical Laboratory Improvement Amendments (CLIA) certified laboratory to do TruSight NGS panel for selected tumors based on the KHCC-MDC recommendations based on the potential to add a clinical benefit to the patients.

In the current study, we aim to evaluate our initial experience, namely, the feasibility of outsourcing molecular testing in terms of the turnaround time from shipment of samples to receiving the results back, the cost of testing, and the importance of integrating the NGS results to reach a diagnosis and/or provide options for targeted treatments.

## Methods

We retrospectively reviewed our KHCC pediatric Neuro-Oncology experience with NGS testing for pediatric CNS tumors between 01/03/2022, and 01/04/2023. We included all patients who were treated at KHCC whom the MDC team recommended to send their tumors for NGS testing and had sufficient RNA quantity for testing.

The decision to send for NGS testing was clinical and based on the MDC discussions and agreement that it could be of a clinical benefit to the patient. “Clinical benefit” could broadly be categorized as either expecting the NGS result to help confirm further the diagnosis when it was challenging to do so by IHC alone or when the radiological images or the clinical course of the patient were not fully aligned with the pathological diagnosis, or when NGS testing was expected to find a targetable alteration based on the pathological diagnosis (e.g., BRAF alterations in low-grade glioma, LGG) that could support the use of a targeted therapy or contribute to predict prognosis. The decisions to send for NGS testing were made either at the time of the initial diagnosis or upon tumor progression. Integration of the NGS results in the patients’ treatment was rediscussed between members of the MDC team and on occasions during KHCC teleconference meetings with SickKids (9), which further helped broaden KHCC’s team knowledge on the implications of these results on the patients’ care.

NGS testing was performed by the CLIA-certified SickKids laboratory. Specimens underwent pathologic evaluation at KHCC, and then scrolls from the formalin-fixed paraffin-embedded blocks were shipped abroad and RNA was extracted at SickKids. TruSight^®^ RNA Pan-Cancer Panel (10) was used, which represents 1,385 genes implicated in cancer pathways. The resulting report was signed by SickKids neuropathologists with recommendations on the implications of the result. Potentially actionable alterations were defined as those, which may result in altered diagnosis, altered treatment, or indicate a germline syndrome. The cost of NGS testing was US$ 1,000 per tumor sample and was covered by the governmental insurance as it was clinically indicated.

In addition, we reviewed the patients’ characteristics, tumor diagnoses, the reason NGS testing was requested, and patient outcome. We assessed the turnaround time and cost needed for this testing in addition to the implications of the results on patients’ care.

## Statistical considerations

This is a descriptive retrospective study to evaluate feasibility. The median and range were used for continuous variables like patients’ characteristics and treatment, whereas counts and percentages were used to present categorical variables. The duration of follow-up was calculated from the time of NGS testing to the patient’s last follow-up date.

This study was approved by the KHCC Institutional Review Board.

## Results

During the study period, 237 patients were discussed in the weekly pediatric Neuro-Oncology MDC meetings (some were discussed more than once). From these, 36 corresponding tumor samples were planned to be sent for NGS testing. Four samples were excluded from this review due to insufficient RNA quantity for testing.

There were 32 patients (14%) eventually who were included, 16 boys and 16 girls. Their median age at the time of NGS testing was 9.5 years (range, 0.9–21.5 years). The median time between tumor biopsy/resection and NGS testing was 2.4 months (range, 0.1–8.8 years). There were 29 brain and three spinal tumors. LGG was diagnosed in 15 tumors (seven were optic pathway gliomas, three were metastatic), 10 were high-grade gliomas (HGG, two were metastatic), and seven were of other histologies (**Table 1**).

**Table 1:** Tumors’ histology with NGS findings, implications on treatment, tumors course, and patients’ outcome.

No.	Tumor histology and location	Timing of NGS testing	NGS findings	NGS result used in treatment/duration of use in months	Tumor course till last follow-up*	Patient outcome/follow-up* (months)
Low-grade glioma						
1	Optic chiasm/hypothalamic ganglioglioma	Diagnosis	**BRAF v600 mutant** No CDK2A deletion	No	Tumor stabilized with chemotherapy	Alive (10.1)
2	Parieto-occipital pediatric-type diffuse LGG	Diagnosis	MYB::PCDHGA1 fusion		Tumor stabilized with chemotherapy	Alive (13.1)
3	Cervico-medullary pediatric-type diffuse LGG	Diagnosis	**KIAA1549::BRAF fusion transcript**	No, parents refused	Tumor progressed on third-line chemotherapy	Alive (9)
4	Brainstem ganglioglioma	Diagnosis	**FGFR1p.N546K,** PTPN11p.E139D, PIK3CAp.V344G, EGFRp.A289T SNVs	No	Tumor stabilized post surgery	Alive (6.7)
5	Suprasellar ganglioglioma	Diagnosis	**BRAF V600E,** CDKN2A- no loss of expression	Yes, dabrafenib (4.7)	Used following progression on chemotherapy (tumor responded)	Alive (6.7)
6	Frontal glioneuronal tumor	Diagnosis	**FGFR1 tyrosine kinase domain ITD**	No	Tumor stabilized with chemotherapy and surgery	Alive (19.5)
7	Metastatic pediatric-type diffuse OPG	Diagnosis	**KIAA1549(exon15)::BRAF(exon9) fusion**	Yes, trametinib (9)	Used following progression on chemotherapy (tumor responded)	Alive (13.5)
8	Metastatic pediatric-type diffuse OPG	Diagnosis	No reportable SNVs/fusions		Tumor progressed on third-line chemotherapy	Dead (11.3)
9	Spinal pediatric-type diffuse LGG	Diagnosis	**KIAA1549(exon15)::BRAF(exon9) fusion**	No	Tumor responded with chemotherapy	Alive (13.5)
10	Optic pathway pilocytic astrocytoma	Progression	**KIAA1549::BRAF fusion transcript**	Yes, trametinib (9.4)	Used following progression on chemotherapy (tumor stabilized)	Alive (9.5)
11	Optic pathway pilocytic astrocytoma	Progression	No reportable SNVs/fusions were detected		Tumor shows slow progression	Alive (6.7)
12	Thalamic pilomyxoid astrocytoma	Progression	NF1p.R1276*SNV		Tumor shows slow progression	Alive (7)
13	Tectal pilocytic astrocytoma	Progression	NF1p.A264Qfs*16 **BRAFp.K601E SNVs**	No	Tumor stabilized with chemotherapy	Alive (13.5)
14	Fronto-temporal DIG	Progression	**BRAFp.G469A**	Yes to control variable cystic tumor response and ascites, dabrafenib, and trametinib (4.3)	Tumor stabilized Ascites controlled	Alive (15.4)
15	Metastatic optic pathway pilocytic astrocytoma	Progression	**KIAA1549(Ex16)::BRAF(E09) fusion**	Yes, trametinib (5.7)	Tumor response	Alive (7)
High-grade glioma						
16	Parietal pediatric-type diffuse HGG	Diagnosis	TP53p.R273C, MSH6p.C687Lfs*10SNVs	Yes, pembrolizumab (5.1)	Tumor stabilized after surgery and focal radiotherapy and pembrolizumab	Alive (5.9)
17	Cerebellar pediatric-type diffuse HGG H3 wild type	Diagnosis	**NRASp.Q61K** NF1p.V33Sfs*9 SNVs	No	Tumor progressed rapidly despite radio-chemotherapy	Dead (10)
18	Frontal pediatric-type diffuse HGG	Diagnosis	MN1 (exon 1 with pseudoexon in-frame insertion)::PATZ1		Tumor stabilized after surgery and focal radiotherapy	Alive (16.8)
19	Frontal embryonal tumor/pediatric-type diffuse HGG IDH-1 wild type	Progression	**EGFR overexpression** PTENp.F341V SNV	No,not accessible	Tumor progressed despite surgeries, radiotherapy and chemotherapy/ASCT	Alive (13)
20	Metastatic thalamic midline glioma H3K27M mutated	Progression	**FGFR1p.K656E** PTENp.F341V	Yes, trametinib (13.7)	Initial response to trametinib then progression	Alive (15.6)
21	Spinal diffuse midline glioma H3K27me3 altered	Diagnosis	**SOX10::NTRK3 fusion**	No	Tumor stabilized after surgery and radio-chemotherapy	Alive (9.4)
22	Spinal glioblastoma (initially diagnosed at 1 year old)	Progression	PTENp.F341V TP53p.H95F SNV		Tumor very slowly progressive	Alive (13.5)
23	Frontal pediatric-type diffuse HGG IDH-1 mutant	Diagnosis	TP53p.P152L IDH1p.R132H **MSH6p.R772W**	Yes, pembrolizumab (11.3)	Initial response then progression	Dead (12.4)
24	Frontal diffuse hemispheric glioma H3 G34 mutant	Progression	P53p.M237l ATRXp.K1361 **IDH1p.R132C** **PIK3R1p.N564D**	No	Tumor progressed rapidly despite radio-chemotherapy	Dead (1.5)
25	Metastatic frontal glioblastoma IDH-1 wild/H3K27 wild	Progression	**SPECC1L(exon11)::NTRK2(exon 14**) fusion transcript	Yes, entrectenib (4.3)	Initial response then progression	Dead (6.5)
Other histologies						
26	Frontal ependymoma	Diagnosis	ZFTA::RELA fusion transcript		Tumor did not recur after surgery and radiotherapy	Alive (10.9)
27	Cervico-medullary mesenchymal tumor (EWSR1 gene rearrangement is positive)	Diagnosis	PTENp.F341V SNV		Tumor stabilized after surgery and radio-chemotherapy	Alive (19.3)
28	Posterior fossa embryonal tumor likely medulloblastoma	Diagnosis	PTENp.F341V SNV		Given intensive chemotherapy/ASCT/focal radiation	Alive (8)
29	Parietal small round blue cell	Diagnosis	No reportable SNVs/fusions		Tumor did not recur after surgery and radio-chemotherapy	Alive (6.8)
30	Parietal astroblastoma grade 2	Diagnosis	PTEN p.F341V NF1p.Y2487		Tumor did not recur after surgery	Alive (13.1)
31	Pineoblastoma	Diagnosis	No reportable SNVs/fusions		Tumor progression	Dead (10.7)
32	Cerebellar undifferentiated round cell sarcoma with BCOR genetic alteration	Progression	**NAB2::STAT6 fusion transcript** P53 p.L194R SNV		Tumor progressed rapidly	Dead (3.1)

DIG, dysembryoplastic ganglioglioma; HGG, high-grade glioma; LGG, low-grade glioma; NGS, next-generation sequencing; OPG, optic pathway glioma.

*Follow up is calculated from the time of sending NGS test to the last follow-up of the patient.

**Figure 1:**
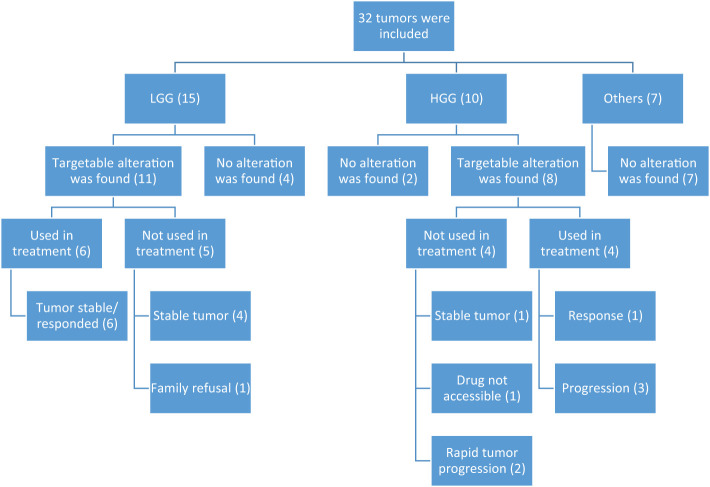
Diagram showing distribution of NGS alterations according to histology and effect on treatment. HGG, high-grade glioma; LGG, low-grade glioma.

The selection of tumors to be tested was made through the MDC team discussions and their expectations of a clinical benefit. Generally, this meant choosing rare diagnoses like a mesenchymal tumor (in patient #27), those challenging to reach a specific diagnosis (in patient #29), or tumors that were felt to have a relatively “unexpected behavior” (like in patients #13 and 14). In addition, we tested some tumors based on the expectation to find an alteration (e.g., BRAF fusion or mutation) and an expectation of a lower response to the traditional chemotherapy protocols (like patients #7 and 8). In summary, the reasons behind recommending NGS testing were either to identify a molecular alteration and assess if a targeted therapy is accessible (20 cases) or to help characterize the tumor more and predict its behavior (12 cases). In 21 cases (66%), NGS testing was performed at the time of the initial diagnosis. There were 19 tumors (59%) that had potentially actionable alterations (**Figure 1**): 14 with FGFR/MAPK pathway inhibitors, two with checkpoint inhibitors, two with NTRK inhibitors, and one with the PI3K inhibitor or IDH1 inhibitor. Two rare BRAF mutations were identified (BRAFp.K601E and BRAFp.G469A) (in patients #13 and 14, respectively). One tumor diagnosed initially as undifferentiated round cell sarcoma harbored NAB2::STAT6 fusion and was reclassified as solitary fibrous tumor (patient #32). This tumor was aggressive and metastatic, and the patient had rapid clinical deterioration. Another tumor (patient #30) initially diagnosed as grade 2 astroblastoma was reclassified as LGG in the absence of MN1 alteration. One tumor could not fit in a specific diagnosis histologically, despite extensive IHC staining, and was diagnosed as small round blue cell tumor and eventually treated as CNS sarcoma (patient #29). NGS did not help characterize this tumor further; however, later, it was diagnosed by DNA methylation as ZFTA ependymoma a with ZFTA: NCOA1/2 fusion (**Table 1**). NGS results in three tumors (patients #4, 16, and 23) suggested an underlying germline syndrome, which was also confirmed by germline testing.

Nine patients (28%) received matched targeted therapy; compassionate dabrafenib/trametinib (6), pembrolizumab (2), and compassionate entrectinib (1). Two patients (# 16 and 23) with biallelic mismatch repair syndrome (BMMRD) had surgical resection then received pembrolizumab during and after radiotherapy without chemotherapy. The remaining seven patients received targeted therapies following tumor progression (they received chemotherapy with or without radiotherapy before). Despite the short duration of using the matched targeted therapy (median 5.7 months, range 4.3–13.7 months), most patients had tumor response, which was sustained when dabrafenib and/or trametinib were used (**Table 1**). There were 10 patients (53%) who did not receive a targeted therapy: six due to stabilization of the tumor with conventional therapies, two due to deterioration in their clinical condition upon tumor progression, one case in which the targeted therapy was not accessible, and one family who preferred to defer the targeted therapy after consuming all options of conventional chemotherapies. During the short follow-up period after NGS testing (median 10.4 months, range 1.5–19.5 months), seven patients died from disease progression including one patient with HGG despite treatment with entrectinib and one patient with BMMRD-associated HGG who received pembrolizumab.

The median turnaround time to receive the NGS result back calculated from the time of shipment was 23.5 calendar days (range, 15–49 days) and from arrival to SickKids was 16 days (range, 8–39 days). Several challenges were encountered during this experience. Some tumor samples were too small to extract sufficient RNA quantity for testing (four tumors), and one tumor sample was lost in shipment; thus, a new sample was sent causing further delay. The experience of utilizing NGS results to help in the diagnosis and treatment of children with CNS tumors was new to the treating team at KHCC, and the test was not yet validated to be performed locally. Accordingly, self-reading and discussion of some NGS results with the SickKids team helped the local team to gain knowledge about the significance of the genomic alterations and the expected response to targeted therapies if any. Access to the targeted therapy was through the compassionate access from Novartis (dabrafenib and trametinib) and Roche (entrectinib), particularly with the lack of pediatric clinical trials at KHCC. Pembrolizumab use was covered through the governmental insurance due to the beneficial evidence of using checkpoint inhibitors in patients with BMMRD (11–13).

## Discussion

Our data demonstrated the feasibility to send out NGS testing in terms of turnaround time and cost for a middle-income country (MIC), with important implications on the diagnosis, treatment, and prognosis for the affected children. Our experience suggests that NGS is not an exclusivity for HIC and our results emphasize the importance of adding molecular diagnostics even in LMICs as an important step to improve the outcome of children with CNS tumors in these countries.

In this initial experience, the selection of the cases was biased toward patients with challenges in diagnosis and/or management. This may explain the high percentage of potentially actionable alterations (59%) reported in this series. In addition, we knew we had access to several special drug access programs with the opportunity to offer some targeted therapies and expect a clinical benefit from the NGS results. In fact, targeted therapy was used in 47% of our patients with an actionable alteration, which constitutes 28% of all tested cases. In the Genome for Kids (G4K) (7), where whole-genome sequencing, whole-exome sequencing, and RNA sequencing were used to test 309 prospectively identified children (85% were newly diagnosed), 86% harbored diagnostic (53%), prognostic (57%), therapeutically relevant (25%), and/or cancer-predisposing (18%) variants. In the MATCH trial (14), where refractory solid tumors, lymphomas, and histiocytic disorders were tested with cancer gene panel sequencing and limited IHC, 109 patients with CNS tumors from the 264 screened (41%) had actionable tumor alterations and 52 patients (48% of those with tumor alteration and 19% of those screened) were enrolled in a trial arm. In this trial, the median turnaround time was 12 days from receiving the sample to completion of testing in this trial, which is shorter compared with ours (16 days).

One would argue that assessing only druggable molecular markers with prognostic value using IHC, FISH, and Sanger sequencing is more realistic in an LMIC setting. This was the conclusion made by Colli et al. (15) from Argentina after they tested 102 pediatric glial and glioneuronal tumors and corelated PFS and OS with several alterations (KIAA1549-BRAF gene fusion, BRAFV600E mutation, H3K27M and H3G34R mutations). While these alterations are the most prevalent, NGS is superior in detecting a wider range of alterations that may change the diagnosis or management. In our experience, two tumor diagnoses were revised based on the NGS finding of NAB2::STAT6 fusion (patient #32) and absence of MN1 alteration (patient #30). In addition, two rare BRAF mutations were identified (BRAFp.G469A, BRAFp.K601E) that would not have been found if a limited test (IHC or FISH) was used to check only for BRAFp.V600E mutation. Furthermore, three tumors (in patients #17, 21, 25) had NRAS and NTRK alterations, respectively, which were unexpected yet targetable alterations. However, even with this wider molecular testing, a proper diagnosis may be difficult to reach without resolving to a more advanced testing, for example DNA methylation profiling, as demonstrated in patient #29. Several studies showed that more extensive testing (e.g., utilizing whole-genome sequencing (WGS), whole-exome sequencing (WES), and RNA sequencing of the tumor) provides clinical data beyond the standard-of-care assays (7, 16).

In the MATCH trial (14),the main reasons for not receiving a targeted treatment for the identified molecular alterations were patients receiving other treatment (32%), poor clinical status (15%), lack of measurable disease (11%), and ineligible diagnosis (10%). The percentage of our patients who did not use a targeted therapy despite having an alteration was similar to the Korean experience (8) (53% vs. 47% respectively) echoing similar reasons, namely, stabilization of tumors, clinical deterioration, or lack of access to the drugs. Practically, these reasons will continue to be the main barriers against performing the tests unless a change in management paradigm is made. The question of whether targeted drugs should be used as a first-line therapy, before conventional chemotherapeutic agents or radiotherapy, is valid especially within the context of the recent FDA approval of the combination of dabrafenib and trametinib as first-line therapy for LGGs and solid tumors with BRAF mutations in children (17). However, this is not easily applicable in countries with limited resources. There is a need for technology transfer and personnel training to establish these molecular tests locally, and a need to have insurance coverage to perform the tests and use of the targeted therapy (1). The high cost of these new targeted drugs remains a significant barrier to their use in LMICs. In fact, this is currently a challenge for our patients with the closure of some special access programs. Efforts to facilitate access to oncology medicines globally and mainly in LMICs were initiated by UICC, the WHO, and Saint Jude Global (18, 19). For this, proper cost-effectiveness studies on the use of targeted therapies in LMICs are needed to balance the current standard of care and poor outcome versus new therapies and their promising results.

Another change in the management paradigm is related to the appropriate timing of performing the molecular tests. Routine upfront testing at the initial cancer diagnosis rather than at tumor progression is more appealing. This may help to better predict the prognosis and allow more time to consider the use of targeted therapies before deterioration in clinical performance occurs. One would argue that the molecular alterations may change with tumor progression and a need for a new biopsy may be preferable in these situations. In addition, the cost–benefit ratio of routine NGS testing needs to be assessed wisely in LMICs settings if access to the targeted drugs is a challenge. It is time to consider MIC participation in molecular clinical trials, considering that 80%–90% of children live in LMICs. Choosing countries with a relatively good infrastructure and centers with trained personnel will not only allow rapid study recruitments and faster results but also enhance the inclusiveness and reduce disparities by allowing wider access to the new technologies and targeted drugs in those communities (1). This should help bridge the survival gap between high- and low-middle-income countries.

In our limited experience, two patients were found to have a cancer predisposition syndrome (namely, BMMRD, patients #16 and 23). This was expected before receiving the NGS results based on their clinical characteristics (consanguinity, family history of cancers, and café au lait spots), and accordingly, a concurrent germline testing was performed, which proved the diagnosis. Several studies that combined tumor and blood NGS testing showed around 7%–18% chance of identifying an underlying cancer predisposition syndrome. This has important consequences on the patient’s care (cancer screening and counseling) and in choosing the treatment approach (e.g., use of checkpoint inhibitors in BMMRD). In addition, one patient with brainstem ganglioglioma and dysmorphic features (patient #4) had PTPN11 alteration in her tumor, which was confirmed on germline testing to have the heterozygous pathogenic mutation leading to a diagnosis of RASopathy spectrum disorder.

We acknowledge some limitations in our study. The main limitation is its retrospective design and the selection bias of choosing tumors to be tested, which was based on the MDC team clinical judgment. The small number of tumors tested and consequent targeted therapies given make it difficult to compare the effectiveness of this approach on tumor control and survival in the absence of a control group. Nevertheless, this is a feasibility experience in a setting of limited similar reports from LMICs. It emphasizes the importance of MDC members’ discussions on how to utilize new cancer advancements selectively. Being a new experience meant we need to move slowly in order to assess the feasibility and appropriateness of sending samples abroad, in terms of turnaround time and cost, and learn how to interpret the results and integrate them into the patients’ care. Moving forward, focused training in molecular pathology was completed by our pathologists and we are now setting the infrastructure to start NGS testing and DNA methylation profiling locally at KHCC, which will have major future implications for our patients. Until then, it is wise to continue to select tumors to be sent abroad for testing, basically tumors with high percentage of expected alterations or tumors that are difficult to diagnose. We would consider the following categories: LGG that are difficult to control by surgical resection and first-line chemotherapy, infant gliomas, HGG, and tumors that are challenging to diagnose by IHC or seem not properly fitting the clinical or radiological picture. Once an alteration is found, the journey of getting access to the drugs starts. It is challenging to find a compassionate access program that will consider applications from LMICs and to consider the shipping challenges as well. Nevertheless, it is worth the journey, and it gets easier with time as the local team gains more expertise and the drug companies’ trust increases with ongoing collaboration with the local team.

In conclusion, we demonstrated the feasibility of sending out NGS testing and the ability to use the results to help in patients’ diagnosis and treatment. However, to achieve this, a close collaboration between pathologists, molecular biologists, and clinicians is needed ideally in a molecular tumor board format. This is most important for CNS tumors with the rapid advancements and integration of the molecular diagnostics now in their classification. In addition, there is a need to convince governments and insurance bodies of the importance of covering these molecular tests and ultimately to approve the targeted therapies to help improve patients’ survival and quality of life.

## Data availability statement

The original contributions presented in the study are included in the article/supplementary material. Further inquiries can be directed to the corresponding author.

## Ethics statement

The studies involving humans were approved by KHCC Institutional Review Board. The studies were conducted in accordance with the local legislation and institutional requirements. The ethics committee/institutional review board waived the requirement of written informed consent for participation from the participants or the participants’ legal guardians/next of kin because the study is retroscpetive in nature and data were de-identified after collection.

## Author contributions

NA: Conceptualization, Validation, Writing – original draft, Writing – review & editing. MA-H: Writing – review & editing. BM: Writing – review & editing. SA: Writing – review & editing. QA: Writing – review & editing. AM: Writing – review & editing. AI: Writing – review & editing. NS: Writing – review & editing. MO: Writing – review & editing. CH: Writing – review & editing. EB: Writing – review & editing.
